# Identification of potential transcriptomic markers in developing pediatric sepsis: a weighted gene co-expression network analysis and a case–control validation study

**DOI:** 10.1186/s12967-017-1364-8

**Published:** 2017-12-13

**Authors:** Yiping Li, Yanhong Li, Zhenjiang Bai, Jian Pan, Jian Wang, Fang Fang

**Affiliations:** 1 Institute of Pediatric Research, Children’s Hospital of Soochow University, Suzhou, China; 2 Department of Nephrology, Children’s Hospital of Soochow University, Suzhou, China; 3 Pediatric Intensive Care Unit, Children’s Hospital of Soochow University, Suzhou, China

**Keywords:** Transcriptomic markers, Co-expression modules, Pediatric sepsis, Diagnosis, Hub genes

## Abstract

**Background:**

Sepsis represents a complex disease with the dysregulated inflammatory response and high mortality rate. The goal of this study was to identify potential transcriptomic markers in developing pediatric sepsis by a co-expression module analysis of the transcriptomic dataset.

**Methods:**

Using the R software and Bioconductor packages, we performed a weighted gene co-expression network analysis to identify co-expression modules significantly associated with pediatric sepsis. Functional interpretation (gene ontology and pathway analysis) and enrichment analysis with known transcription factors and microRNAs of the identified candidate modules were then performed. In modules significantly associated with sepsis, the intramodular analysis was further performed and “hub genes” were identified and validated by quantitative real-time PCR (qPCR) in this study.

**Results:**

15 co-expression modules in total were detected, and four modules (“midnight blue”, “cyan”, “brown”, and “tan”) were most significantly associated with pediatric sepsis and suggested as potential sepsis-associated modules. Gene ontology analysis and pathway analysis revealed that these four modules strongly associated with immune response. Three of the four sepsis-associated modules were also enriched with known transcription factors (false discovery rate-adjusted *P* < 0.05). Hub genes were identified in each of the four modules. Four of the identified hub genes (MYB proto-oncogene like 1, killer cell lectin like receptor G1, stomatin, and membrane spanning 4-domains A4A) were further validated to be differentially expressed between septic children and controls by qPCR.

**Conclusions:**

Four pediatric sepsis-associated co-expression modules were identified in this study. qPCR results suggest that hub genes in these modules are potential transcriptomic markers for pediatric sepsis diagnosis. These results provide novel insights into the pathogenesis of pediatric sepsis and promote the generation of diagnostic gene sets.

**Electronic supplementary material:**

The online version of this article (10.1186/s12967-017-1364-8) contains supplementary material, which is available to authorized users.

## Background

Sepsis represents a complex disease with the dysregulated inflammatory response and high mortality rate. It is the world’s leading killer of children [1]. However, current knowledge of the pathogenesis of sepsis is limited [2, 3].

In the past decade, several studies have reported the transcriptional profiling of sepsis using microarrays to identify candidate genes involved in sepsis development [4–7]. Co-expression module analysis of transcriptomic dataset has the likelihood of discovering robust candidates for diagnosis and treatment. Therefore, we investigated gene expression patterns between pediatric sepsis patients and healthy controls in this study based on public microarray dataset. Network construction and module detection were performed. The importance of candidate modules identified in this study were evaluated, and modules most significantly associated with sepsis were further interpreted by enrichment analysis, intramodular analysis and quantitative real-time PCR (qPCR).

To carry out these analyses, we used the R software (v3.3.2) [8] and Bioconductor packages [9] for data pre-procession and weighted gene co-expression network analysis. Functional interpretation and network construction of co-expression modules were also performed using DAVID [10, 11] and Cytoscape [12] software, respectively. Enrichment analysis of the candidate module genes with known transcription factors and microRNAs was also performed using WebGestalt [13]. Validation of gene expression patterns was performed by qPCR in this study.

## Methods

### Microarray datasets search and selection

In this study, we searched public microarray datasets till Jul 12, 2016, according to the keywords “sepsis” in Gene Expression Omnibus (GEO) database [14]. The datasets obtained were further selected for subsequent analysis, and our selection criteria were: (a) case–control dataset; (b) dataset using whole blood from children for gene expression analysis; (c) dataset providing detailed gene expression data; (d) dataset with sample size (septic children and controls in total) larger than 100. Animal studies and studies of adults were excluded.

### Pre-procession of microarray gene expression dataset

One dataset fulfilled the selection criteria and was used for further analysis. This eligible dataset (GSE13904) was generated using the Affymetrix Human Genome U133 Plus 2.0 Array from 99 pediatric sepsis patients (32 sepsis and 67 septic shock patients) and 18 normal controls [7]. Raw data saved in.CEL files of the eligible dataset was downloaded from GEO database, and then pre-processed (background correction, quantile normalization, log2 transformed) using the Robust Multichip Average (RMA) method of the R package “affy” [15]. Next, the hybridization probes were mapped to genes (Entrez IDs) according to the platform table. Probes mapping to multiple genes and probes not mapping to genes were excluded. When multiple probes mapped to the same gene, arithmetic mean of probe values were calculated to represent gene expression.

### Weighted gene co-expression network analysis

Weighted gene co-expression network analysis was carried out using the R package “WGCNA” [16] in this study. Pre-processed gene expression data were first checked for missing values and outliers. The genes and samples which passed the test were collected for network analysis. One-step network construction and module detection were then performed, with the soft-thresholding power β set to 14 according to the criterion of approximate scale-free topology (Additional file 1), the minimum module size set to 30, and the threshold for merging of modules set to 0.25. Modules significantly associated with sepsis were identified based on the correlation between module eigengenes and sample types (sepsis patients versus healthy controls). Gene relationships to sepsis and modules were then evaluated by gene significance (GS, correlation of individual gene expression with sepsis) and module membership (MM, correlation of individual gene expression with module eigengene). Network construction of co-expression modules was also performed using Cytoscape 3.4.0 [12] software.

### Enrichment analysis

Functional interpretation [gene ontology (GO) analysis and Kyoto Encyclopedia of Genes and Genomes (KEGG) pathway analysis] of the co-expression genes in sepsis-associated modules was further performed using DAVID 6.8 [10, 11]. In GO analysis, a *P* value threshold of 0.05 was used to identify significantly enriched GO terms [17]. In pathway analysis, enrichment analysis was carried out using the hypergeometric test with a *P* value threshold of 0.05 based on the KEGG database [18]. Enrichment analysis of the candidate module genes with known transcription factors and microRNAs was also performed using WebGestalt [13], according to the criteria: (a) false discovery rate (FDR)-adjusted *P* value < 0.05; (b) a minimum number of genes in a category: two.

### Intramodular analysis and quantitative real-time PCR

For modules significantly associated with sepsis, the intramodular analysis was performed and “hub genes” were identified according to the criteria: (a) LOG10 (*P* value of GS) ≥ 10; (b) MM ≥ 0.8.

Validation of hub gene expression patterns was performed by qPCR in this study. 45 pediatric sepsis patients were included. 16 children who were scheduled for minor elective surgery such as circumcision or inguinal hernia repair were also included as the control group. All heparinized blood samples were obtained from Children’s Hospital of Soochow University. Informed consent was obtained from each participating individual’s guardian. The study procedure was approved by the ethics committee of Children’s Hospital of Soochow University.

Mononuclear cells (MNCs) were isolated, and then stored at − 80 °C before RNA extraction. Total RNA was extracted using RNAiso (TaKaRa, Dalian, China). The RNA was reverse-transcribed using oligo-dT, and mouse mammary tumor virus reverse transcriptase. qPCR was performed with SYBR Green master mix. Primers designed were shown in Additional file 2. Gene expression was normalized to β-actin mRNA. The relative expression of gene transcript was calculated using the 2^−ΔΔCt^ method. Comparison of clinical characteristics between study groups was performed using the Mann–Whitney U test for continuous variables and the Fisher’s exact test for categorical variables. Mann–Whitney U test was also performed to determine the expression difference between septic children and the control group. Statistical analyses were performed with GraphPad Prism software (GraphPad Software Inc.). All *P* values are two-sided. *P* < 0.05 was considered as statistically significant.

In addition, the diagnostic performance of hub genes were also evaluated in the validation group (45 septic children and 16 controls) by receiver operating characteristic (ROC) curve plotting and area under the curve (AUC) values calculation using the R package “pROC” [19].

## Results

### Co-expression modules in pediatric sepsis development

**Fig. 1:**
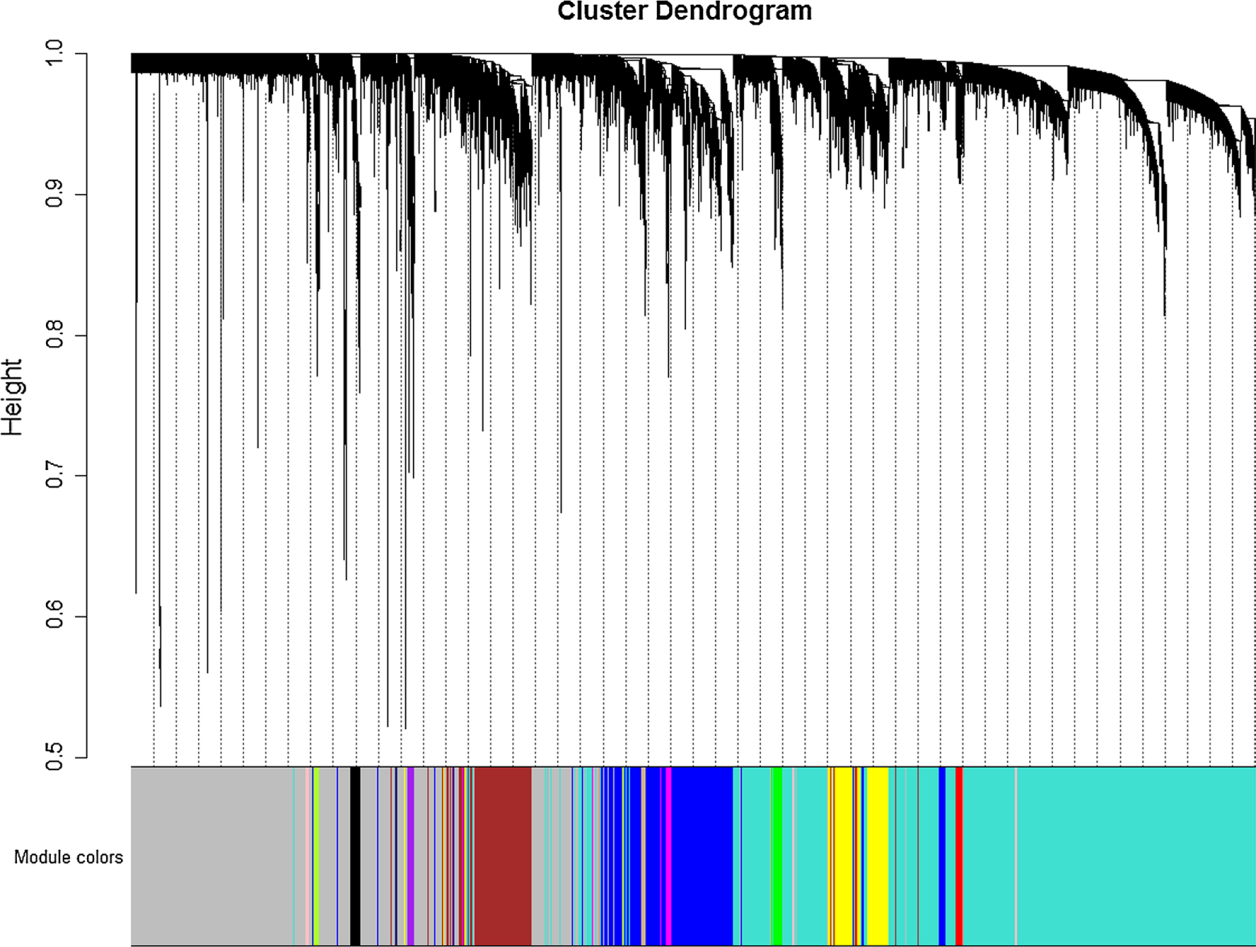
Hierarchical clustering dendrogram used for module identification

**Fig. 2:**
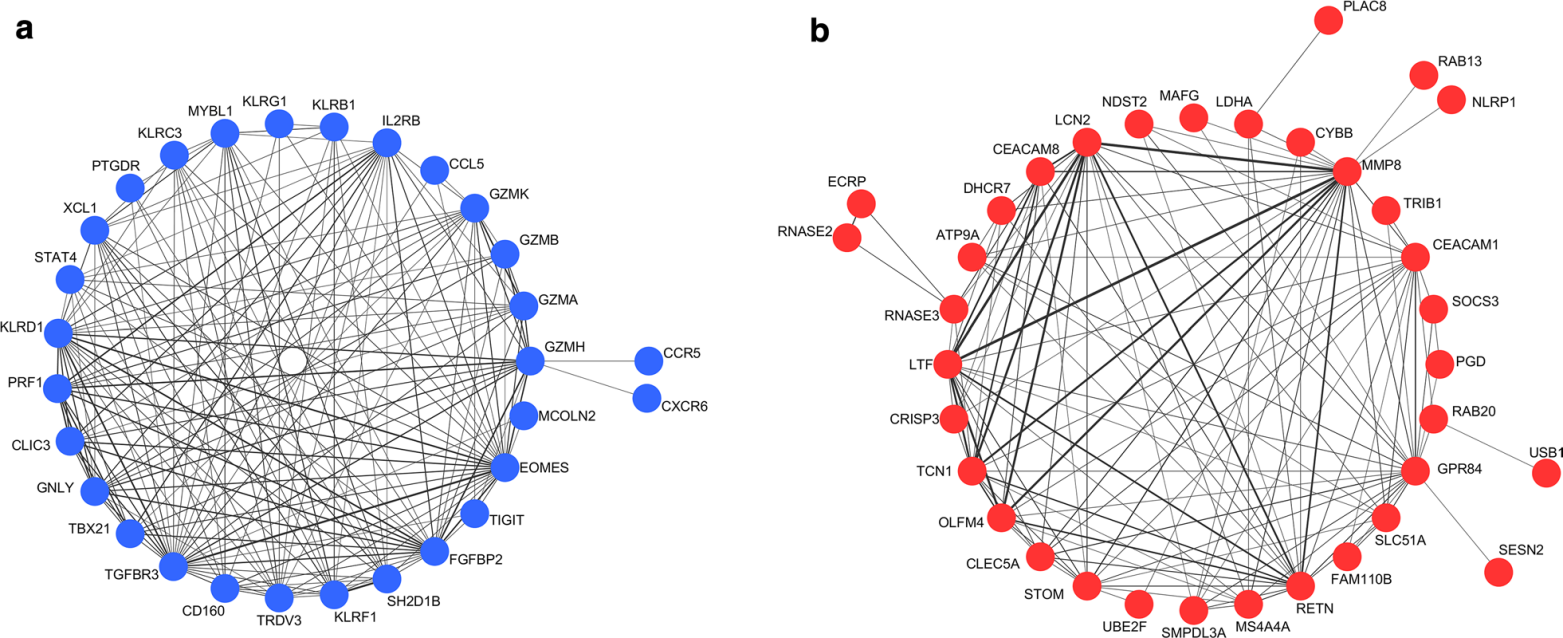
Co-expression networks of the top 2 sepsis-associated modules. **a** Network of module “midnight blue”. **b** Network of module “cyan”. Edge widths are proportional to the correlation coefficients

Original search identified one eligible microarray dataset (GSE13904) [7]. Pre-processing of this dataset resulted in expression data of 20,464 genes in 99 pediatric sepsis samples and 18 normal controls. According to the parameters (soft-thresholding power β = 14, minimum module size = 30, and the threshold for merging of modules = 0.25) and the hierarchical clustering dendrogram (Fig. 1) used for the module identification in this study, 15 modules in total were detected with sizes ranging from 8359 to 39 genes. Among the 15 candidate modules, four modules (module “midnight blue”, module “cyan”, module “brown”, and module “tan”) were most significantly associated with sepsis and suggested as potential sepsis-associated modules (see Table 1). Co-expression networks of the top 2 sepsis-associated modules (module “midnight blue” and module “cyan”) are shown in Fig. 2.

**Table 1:** Top 4 sepsis-associated co-expression modules

Module	Correlation coefficient with pediatric sepsis	*P* ^a^	Number of genes
Midnight blue	− 0.67	2 × 10^−16^	39
Cyan	0.65	2 × 10^−15^	39
Brown	0.54	4 × 10^−10^	1382
Tan	− 0.53	1 × 10^−9^	80

^a^Student asymptotic *P* value for correlation

### Enrichment analysis results

Advanced analyses (GO analysis and pathway analysis) were carried out for further functional investigation of the four sepsis-associated co-expression modules. Table 2 presented a summary of the GO and pathway analysis results. In the GO analysis, the top GO biological process terms enriched are “cellular defense response” in module “midnight blue”, “innate immune response” in module “cyan”, “inflammatory response” in module “brown”, and “antigen processing and presentation of peptide or polysaccharide antigen via MHC class II” in module “tan”. In the pathway analysis, the most significant pathways identified were natural killer cell-mediated cytotoxicity in module “midnight blue”, osteoclast differentiation in module “brown”, and asthma in module “tan”, when we mapped the module genes to the KEGG database. No significantly enriched pathway was identified in module “cyan”.

**Table 2:** Summary of the GO and pathway analysis results of the four sepsis-associated modules

Module	GO (BP)			GO (CC)			GO (MF)			KEGG pathway		
	Term	Genes	*P*	Term	Genes	*P*	Term	Genes	*P*	Term	Genes	*P*
Midnight blue	Cellular defense response	6	1.59 × 10^−7^	External side of plasma membrane	4	7.73 × 10^−3^	Carbohydrate binding	5	4.62 × 10^−4^	Natural killer cell mediated cytotoxicity	4	4.43 × 10^−3^
Cyan	Innate immune response	5	9.49 × 10^−3^	Extracellular space	12	4.40 × 10^−5^	Ribonuclease activity	2	4.12 × 10^−2^	NA	NA	NA
Brown	Inflammatory response	68	1.05 × 10^−10^	Extracellular exosome	346	3.36 × 10^−24^	Protein binding	810	2.55 × 10^−11^	Osteoclast differentiation	43	1.90 × 10^−12^
Tan	Antigen processing and presentation of peptide or polysaccharide antigen via MHC class II	6	8.89 × 10^−10^	MHC class II protein complex	6	3.36 × 10^−9^	MHC class II receptor activity	5	1.17 × 10^−7^	Asthma	6	8.95 × 10^−8^

*GO* gene ontology, *BP* biological process, *CC* cellular component, *MF* molecular function, *KEGG* Kyoto Encyclopedia of Genes and Genomes, *NA* not available

**Fig. 3:**
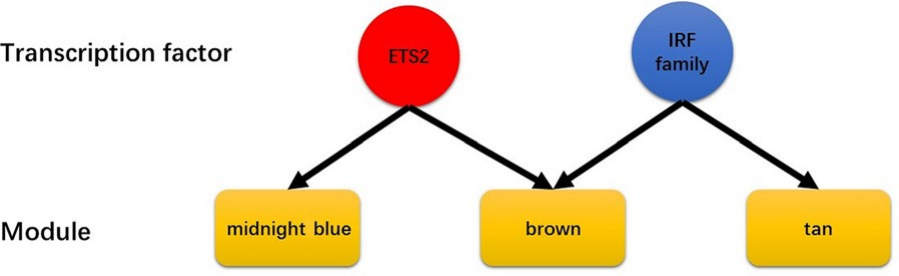
Enriched transcription factors of particular interest. ETS2 and the IRF family were identified as regulators of multiple sepsis-associated co-expression modules

Enrichment analysis of genes in the four sepsis-associated modules with known transcription factors and microRNAs was also performed using WebGestalt [13]. As presented in Table 3, three of the four sepsis-associated modules were enriched with known transcription factors (FDR-adjusted *P* value < 0.05). Among them, transcription factors of particular interest are ETS proto-oncogene 2, transcription factor (ETS2) and the interferon regulatory transcription factor (IRF) family, as they function in the regulation of multiple sepsis-associated co-expression modules (see Fig. 3). However, no significantly enriched microRNA was identified in this study.

**Table 3:** Summary of the transcription factor enrichment analysis results of the four sepsis-associated modules

Module^a^	Transcription factor	Official full name	FDR-adjusted *P* value
Midnight blue	ETS2	ETS proto-oncogene 2, transcription factor	1.64 × 10^−2^
Brown^b^	ETS2	ETS proto-oncogene 2, transcription factor	5.85 × 10^−8^
	ELF1	E74 like ETS transcription factor 1	9.52 × 10^−6^
	SPI1	Spi-1 proto-oncogene	4.37 × 10^−5^
	ETV4	ETS variant 4	3.86 × 10^−4^
	RUNX1	Runt related transcription factor 1	1.37 × 10^−3^
	SRF	Serum response factor	1.37 × 10^−3^
	GABPA	GA binding protein transcription factor alpha subunit	2.51 × 10^−3^
	JUN	Jun proto-oncogene, AP-1 transcription factor subunit	3.07 × 10^−3^
	ETV7	ETS variant 7	3.07 × 10^−3^
	IRF family	Interferon regulatory transcription factor family	8.03 × 10^−3^
	SREBF1	Sterol regulatory element binding transcription factor 1	9.52 × 10^−3^
Tan	IRF1	Interferon regulatory factor 1	4.03 × 10^−2^
	POU5F1	POU class 5 homeobox 1	4.03 × 10^−2^
	POU2F1	POU class 2 homeobox 1	4.03 × 10^−2^

*FDR* false discovery rate

^a^No significantly enriched transcription factor was identified in module “cyan”

^b^Transcription factors with FDR-adjusted *P* value < 0.01 were listed for module “brown” for the sake of brevity

### Hub gene identification and validation

**Fig. 4:**
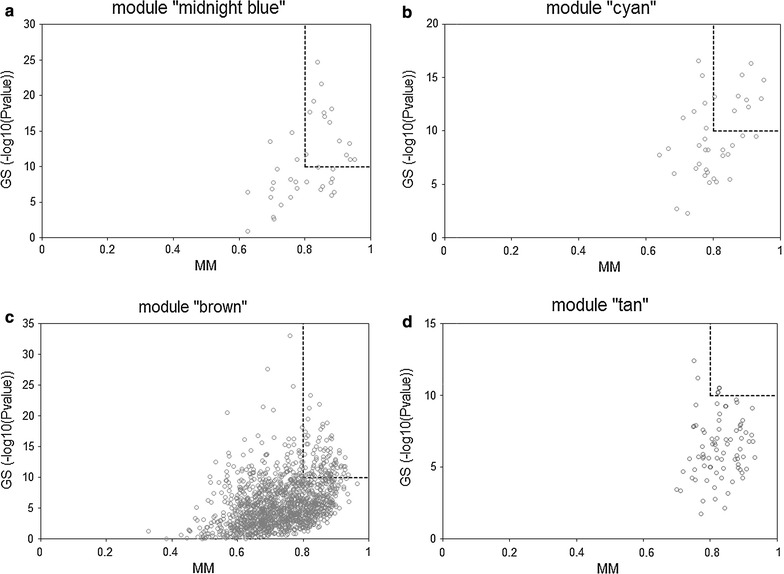
Scatterplots of the intramodular analysis results and hub genes identified in the four sepsis-associated modules. **a** Scatterplot for module “midnight blue”. **b** Scatterplot for module “cyan”. **c** Scatterplot for module “brown”. **d** Scatterplot for module “tan”. *GS* gene significance, *MM* module membership

**Fig. 5:**
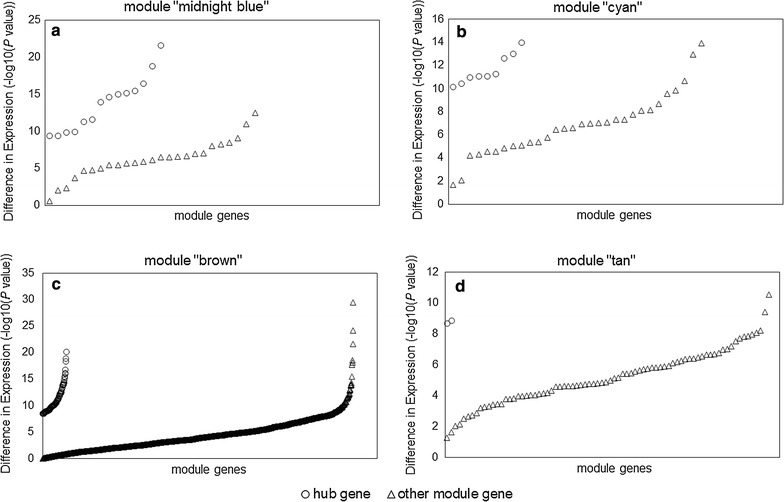
Differences in expression of hub genes and other module genes between 99 pediatric sepsis samples and 18 normal controls from dataset GSE13904. **a** Differences in expression for module “midnight blue”. **b** Differences in expression for module “cyan”. **c** Differences in expression for module “brown”. **d** Differences in expression for module “tan”

**Fig. 6:**
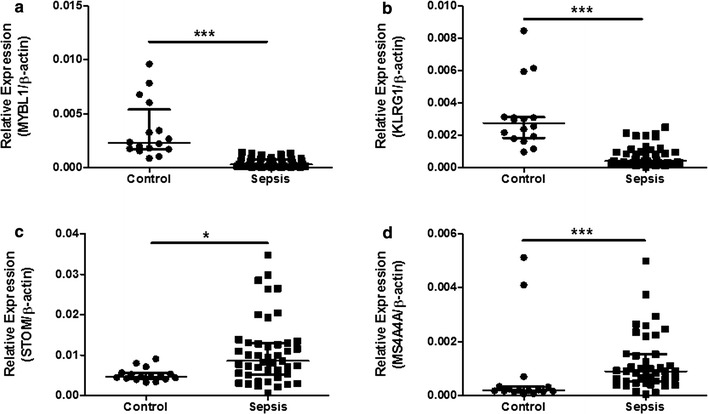
qPCR results of *MYBL1*, *KLRG1*, *STOM* and *MS4A4A* between 45 septic children and 16 controls. **a** *MYBL1* relative expression comparison between pediatric sepsis patients and controls (Mann–Whitney U test, ***P < 0.001). **b** *KLRG1* relative expression comparison between pediatric sepsis patients and controls (Mann–Whitney U test, ***P < 0.001). **c** *STOM* relative expression comparison between pediatric sepsis patients and controls (Mann–Whitney U test, *P = 0.04). **d** *MS4A4A* relative expression comparison between pediatric sepsis patients and controls (Mann–Whitney U test, ***P < 0.001)

**Fig. 7:**
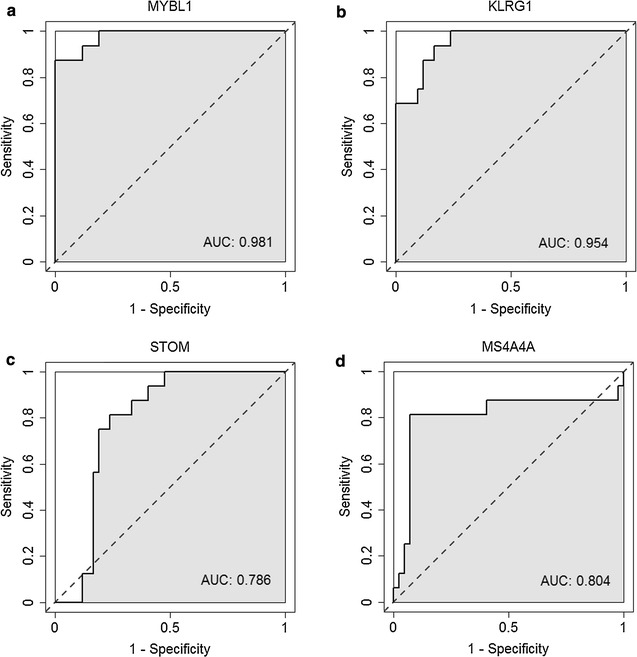
Receiver operating characteristic (ROC) curves of the hub genes on diagnosis of pediatric sepsis. AUC: area under the ROC curve. **a** ROC curve of *MYBL1*. **b** ROC curve of *KLRG1*. **c** ROC curve of *STOM*. **d** ROC curve of *MS4A4A*

The intramodular analysis was performed in the four sepsis-associated co-expression modules, and hub genes were identified in each of the four modules. As shown in Fig. 4, 14, 9, 98, and two hub genes were identified within module “midnight blue”, “cyan”, “brown”, and “tan” respectively. Significant changes in expression of those hub genes were detected between 99 pediatric sepsis samples and 18 normal controls from dataset GSE13904 [7] (Fig. 5). four of the identified hub genes [MYB proto-oncogene like 1 (*MYBL1*) and killer cell lectin-like receptor G1 (*KLRG1*) form module “midnight blue”, stomatin (*STOM*) and membrane spanning 4-domains A4A (*MS4A4A*) from module “cyan”], predicted to be potential biomarkers for pediatric sepsis in intramodular analysis and with little known in sepsis, were further assessed for their difference in expression between 45 septic children and 16 controls (Table 4) by qPCR. As presented in Fig. 6, the expression levels of *MYBL1* and *KLRG1* in the sepsis group were significantly lower than those of the control group (*P* < 0.001 respectively, see Fig. 6a, b). While *STOM* and *MS4A4A* were significantly overexpressed in sepsis samples, compared with controls (*P* = 0.04 and *P* < 0.001 respectively, see Fig. 6c, d). As for diagnostic prediction quality, the four hub genes showed good performance as well according to the ROC analysis in the validation group (45 septic children and 16 controls) (see Fig. 7). Both the qPCR results and ROC analysis results suggest that the four hub genes (*MYBL1*, *KLRG1*, *STOM* and *MS4A4A*) could be novel diagnostic biomarkers for pediatric sepsis.

**Table 4:** Clinical characteristics of the validation group

Characteristic	Sepsis	Control	*P*
Number	45	16	
Age, median years [range]	1.50 [0.08–13.42]	1.92 [0.08–10.50]	0.95^a^
Gender			0.22^b^
Male	27	13	
Female	18	3	
Infection site			
Lung (%)	15 (33.3)	–	–
Brain (%)	11 (24.4)	–	–
Others (%)	19 (42.2)	–	–
Septic shock (%)	17 (37.8)	–	–
ICU stay, median days [range]	5.38 [0.08, 30.00]	–	–
ICU mortality (%)	13(28.9)	–	–

^a^ *P* value of the Mann–Whitney U test

^b^ *P* value of the Fisher’s exact test

## Discussion

Some genes have been reported to be up-regulated or down-regulated in pediatric sepsis patients [5, 7]. Identification of the most important candidate genes and pathways involved in sepsis pathogenesis is a challenge currently. Growing high-throughput transcriptomic data enables weighted gene co-expression network analysis of microarray datasets which has the likelihood of discovering robust candidates for diagnosis and treatment. Hence in this study, we performed a weighted gene co-expression network analysis of public microarray dataset to identify potential transcriptomic markers in developing pediatric sepsis.

In this analysis, 15 co-expression modules were identified, among which four modules (module “midnight blue”, module “cyan”, module “brown”, module “tan”) were significantly associated with pediatric sepsis and were potential sepsis-associated modules. The enrichment analysis indicated that transcription factors (ETS2 and the IRF family) play roles in the regulation of multiple sepsis-associated modules. So far there is increasing evidence that the IRF family plays a part in sepsis [20, 21]. Whereas little is known about the impact of ETS2 on sepsis and further investigation is needed.

Through intramodular analysis, 123 hub genes in total were identified in the four sepsis-associated co-expression modules, including hub genes known to play roles in sepsis, and hub genes without previous studies in sepsis. Among those novel hub genes, we assessed the expression patterns of *MYBL1*, *KLRG1*, *STOM* and *MS4A4A* from the top 2 sepsis-associated modules (module “midnight blue” and module “cyan”) by qPCR. Significantly different expressions between pediatric sepsis patients and controls were detected for all four genes, validating the intramodular analysis results. *MYBL1* belongs to the MYB family and is involved in adenoid cystic carcinoma and pediatric glioma [22, 23]. *KLRG1* encodes a receptor on antigen-experienced T cells and natural killer cells [24, 25]. It is suggested as a senescent marker of human T cells [26]. In tumor microenvironment, *KLRG1* is significantly overexpressed in T cells [27]. *STOM* encodes a major lipid-raft protein stomatin, which locates at the plasma membrane of multiple cell types [28–30], and is associated with non-small cell lung cancer [31] and erb-b2 receptor tyrosine kinase 2-positive breast cancer [32]. MS4A4A, a member of the membrane-spanning 4-domains subfamily A, is reported to be a cell-surface marker of plasma cells and M2 macrophages [33]. It is also up-regulated in the autopsied brain tissue of Alzheimer’s disease patients [34]. In this study, ROC analysis results further indicate that the four hub genes (*MYBL1*, *KLRG1*, *STOM* and *MS4A4A*) had good diagnostic performance in sepsis, close to that of genes previously reported [35]. Although the exact contributions of the four and other novel hub genes to sepsis are not clear yet, further research is necessary as those genes could be potential transcriptomic markers for sepsis.

Our analysis also has some limitations. The first limitation is the insufficient sample size. A second limitation is the lack of subgroup analyses based on potential influential factors, including age, sex, disease severity and platform usage, considering the reported impact of gender and age on pediatric sepsis patients [36]. The third limitation is the incomplete biological knowledge base and pathway information available at present. Hence, to achieve a more convincible conclusion, further analysis using larger sample size is required. Stratified analyses on different factors such as age, gender, disease severity, and platform usage are also needed. Functional studies should be performed as well to address the exact roles of the candidate hub genes in pediatric sepsis.

## Conclusions

In conclusion, we identified four candidate co-expression modules that were differentially expressed between pediatric sepsis patients and normal controls. GO, and pathway analyses revealed that those candidate modules strongly associated with immune response. Transcription factors associated with the modules were also identified through enrichment analysis in this study. qPCR results suggest hub genes (*MYBL1*, *KLRG1*, *STOM* and *MS4A4A*) in the candidate modules as promising potential transcriptomic markers for pediatric sepsis diagnosis. To the best of our knowledge, there is no reported weighted gene co-expression network analysis for sepsis so far. We hope this study can help in the diagnosis and treatment of pediatric sepsis.

## Additional files

**Additional file 1.** Analysis of network topology for candidate soft-thresholding powers (βs).

**Additional file 2.** Primers designed for validation of hub gene expression patterns by qPCR.

## Abbreviations

AUC: area under the curve
BP: biological process
CC: cellular component
ETS2: ETS proto-oncogene 2, transcription factor
FDR: false discovery rate
GEO: Gene Expression Omnibus
GO: gene ontology
GS: gene significance
ICU: Intensive Care Unit
IRF: interferon regulatory transcription factor
KEGG: Kyoto Encyclopedia of Genes and Genomes
KLRG1: killer cell lectin-like receptor G1
MF: molecular function
MM: module membership
MNCs: mononuclear cells
MS4A4A: membrane spanning 4-domains A4A
MYBL1: MYB proto-oncogene like 1
NA: not available
qPCR: quantitative real-time PCR
RMA: Robust Multichip Average
ROC: receiver operating characteristic
STOM: stomatin
