# Supplementary material for: Identification of potential transcriptomic markers in developing pediatric sepsis: a weighted gene co-expression network analysis and a case–control validation study
Source: J Transl Med. 2017 Dec 13;15:254. doi: 10.1186/s12967-017-1364-8 (PMC5729245; doi:10.1186/s12967-017-1364-8)

Additional file 1: Analysis of network topology for candidate soft-thresholding powers (βs). β was set to 14, the lowest power for which the scale-free topology fit index curve flattened out upon reaching close to 0.90.

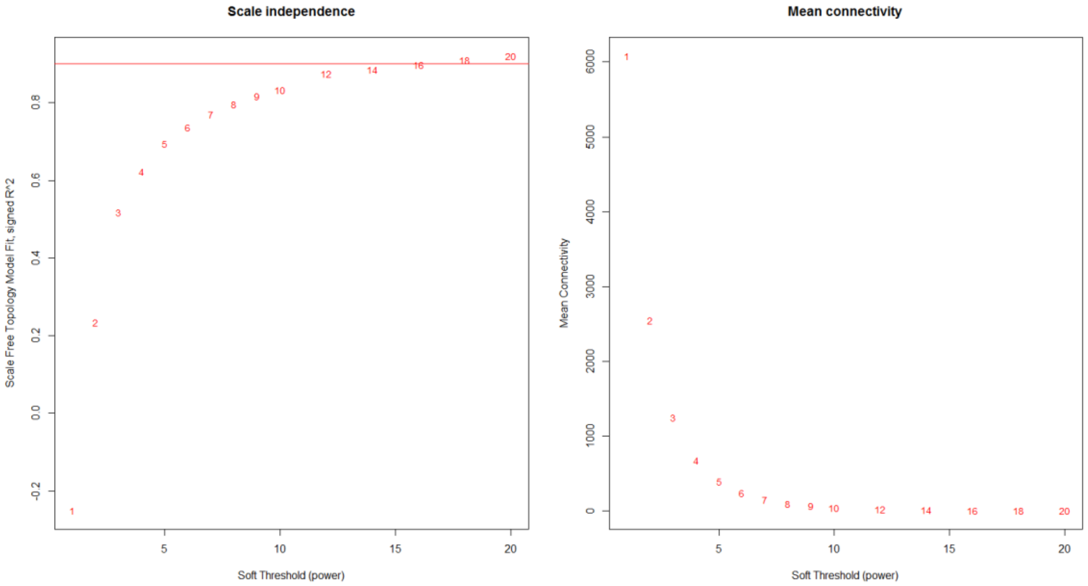

Supplement: Supplementary file 1 — Additional file 1. Analysis of network topology for candidate soft-thresholding powers (βs). [file 12967_2017_1364_MOESM1_ESM.pdf]
