# Supplementary material for: Identification of potential transcriptomic markers in developing pediatric sepsis: a weighted gene co-expression network analysis and a case–control validation study
Source: J Transl Med. 2017 Dec 13;15:254. doi: 10.1186/s12967-017-1364-8 (PMC5729245; doi:10.1186/s12967-017-1364-8)
Supplement: Supplementary file 2 — Additional file 2. Primers designed for validation of hub gene expression patterns by qPCR. [file 12967_2017_1364_MOESM2_ESM.pdf]

Additional file 2. Primers designed for validation of hub gene expression patterns by qPCR.

**Primer**		**Sequence (5' – 3')**
MYBL1	Sense	AGGCAAGCAGTGTAGAGAAAGA
	Antisense	CGATTTCCCAACCGCTTATGT
KLRG1	Sense	CCAGACCGCTGGATGAAATATG
	Antisense	CTGATTGTCCGTTATCACAAGGA
STOM	Sense	CACACACGGGACTCCGAAG
	Antisense	ATGAGAACGCCACCAAAATCC
MS4A4A	Sense	TGAGCCTTAGCATGGGAATAACA
	Antisense	CCCGATATACACGGAAATAGGGT
β-actin	Sense	AGAGCTACGAGCTGCCTGAC
	Antisense	AGCACTGTGTTGGCGTACAG
